# Urinary tract infection caused by rare yeast *Vanrija humicola* in a urothelial carcinoma patient: a case report and literature review

**DOI:** 10.3389/fimmu.2026.1845780

**Published:** 2026-06-09

**Authors:** Hanman Qiu, Hong Du, Xiaohui Li, Xunsong Wang, Yan Li, Hazrat Bilal, Bin Xu

**Affiliations:** 1 School of Public Health, Jiangxi Medical College, Nanchang University, Nanchang, Jiangxi, China; 2 Jiangxi Key Laboratory of Oncology (2024SSY06041), Jiangxi Cancer Hospital & Institute, The Second Affiliated Hospital of Nanchang Medical College, Nanchang, Jiangxi, China; 3 Department of Pharmacy, Qingdao Mental Health Center, Qingdao, Shandong, China; 4 Jiangxi Key Laboratory of Translational Cancer Research, Jiangxi Cancer Hospital, The Second Affiliated Hospital of Nanchang Medical College, Jiangxi Cancer Institute, Nanchang, Jiangxi, China

**Keywords:** antifungal susceptibility testing, case report, opportunistic rare fungal infection, urothelial carcinoma, *Vanrija humicola*

## Abstract

**Background:**

Urinary tract infections from rare fungi are rising in immunocompromised cancer patients. Early diagnosis and antifungal testing of *Vanrija humicola* are essential to guide treatment and improve outcomes in urothelial carcinoma.

**Case description:**

An 82-year-old man with advanced urothelial carcinoma and metastases to the bone and lungs was admitted to Jiangxi Cancer Hospital on September 22, 2025. He presented with urinary frequency, urgency, dysuria, and pain in the left hip. At admission, he was started on cefoperazone–sulbactam for a suspected urinary tract infection. On September 29, he underwent transurethral resection of the prostate. Pathology confirmed a malignant bladder tumor (urothelial carcinoma). Palliative radiotherapy and chemo-immunotherapy with toripalimab, gemcitabine, and cisplatin were initiated on October 10. Urine culture performed on October 10 grew *Enterococcus faecalis*. An unidentified fungal culture obtained on October 12, which was confirmed as *Vanrija humicola* by ITS and D1–D2 rDNA sequencing. Antifungal susceptibility showed voriconazole MIC 0.12 mg/L, fluconazole 4 mg/L, amphotericin B <1 mg/L, echinocandins >8 mg/L. He was treated with intravenous voriconazole and amoxicillin–clavulanate, with close clinical monitoring. During treatment, his urinary symptoms gradually resolved, inflammatory markers returned to normal levels, and the pain in his left hip improved. The patient was discharged on October 22, 2025.

**Conclusions:**

This case shows the opportunistic pathogenic potential of *V. humicola* in immunocompromised patients. Accurate identification and antifungal testing are essential, and clinicians should consider rare fungi for timely, effective treatment.

## Introduction

Bladder cancer ranks as the ninth most common cancer worldwide, and its incidence continues to increase globally (1). Urothelial carcinoma is the most common histological type of bladder cancer, and cigarette smoking is a well-known major risk factor (2). Urothelial carcinoma of the bladder (UCB) is more common in men than in women, with a ratio of about 3–4:1. The highest number of cases occurs in people aged 85–89, while mortality rates are higher in those over 95 years of age (3). Transurethral resection of bladder tumors (TURBT) is the main method used to diagnose and stage bladder cancer. Treatment usually involves platinum-based chemotherapy and immunotherapy (4, 5). Bladder cancer patients are also at high risk of secondary infections because of invasive medical interventions such as catheterization and surgery. About 22% of patients develop urinary tract infections (UTIs) after bladder tumor surgery (6).

UTIs are mainly caused by Gram-negative bacteria, with *Escherichia coli* being the most frequent pathogen (7, 8). Recently, fungal infections have also become increasingly common in immunocompromised cancer patients (9). Studies indicate that fungi can significantly influence the tumor microenvironment, affecting cancer development and progression (10). In cancer patients, *Candida* and *Aspergillus* species remain the leading causes of invasive fungal infections (IFIs), with an increasing incidence of rare fungal species being reported (11). Infections caused by rare fungi are challenging to diagnose and may delay the initiation of targeted antifungal therapy (12).

*Vanrija humicola* (formerly *Cryptococcus humicola*) is a basidiomycetous yeast within the family Trichosporonaceae and the order Trichosporonales, a group phylogenetically related to Trichosporon species (13). These environmental yeasts are considered opportunistic pathogens, often colonizing catheters in immunocompromised patients and causing UTIs following urinary tract instrumentation (14). Here, we report a case of UTI caused by *V. humicola* in a patient with UCB. This report highlights the pathogenic potential of this rare opportunistic fungus in high-risk populations and discusses its diagnosis, treatment, and possible sources of infection.

## Case presentation

The patient is an 82-year-old male with a history of coronary heart disease. He underwent coronary artery bypass grafting (CABG) 5 years ago and has been on long-term antiplatelet and lipid-lowering therapy (clopidogrel, aspirin, atorvastatin). He had a 30-year history of heavy smoking (three packs/day) but quit over 20 years ago. He reported no alcohol use or family history of cancer.

He complained of frequent, urgent, and painful urination accompanied by pain in the left hip joint and thigh for more than one month. Oral analgesics taken by the patient provided no relief, and he presented to a municipal local hospital on September 20, 2025. MRI revealed significant prostatic enlargement and a soft-tissue mass in the left iliac bone; however, no treatment was administered.

Subsequently, the patient visited Jiangxi Cancer Hospital on September 22, 2025. On admission, his vital signs were stable (temperature 37.1 °C, heart rate 104 bpm, blood pressure 133/65 mmHg). Physical examination was unremarkable, with no palpable lymphadenopathy and normal cardiovascular, respiratory, abdominal, and neurological findings. Chest and abdominal CT, along with a whole-body bone scan, revealed a subpleural nodule in the right middle lobe. Scattered small nodules were also seen in both lungs. In addition, osteolytic bone destruction was detected, with abnormal radionuclide uptake in the left ilium, acetabulum, and ischium, accompanied by a localized soft-tissue mass. Urinalysis showed gross hematuria, marked elevations in protein, red blood cells (RBCs), and white blood cells (WBCs). Based on these findings, the clinicians initially suspected prostate cancer with pulmonary and bone metastases, complicated by a UTI. Empiric intravenous anti-infective therapy with cefoperazone–sulbactam was then initiated at a dose of 3.0 g every 12 hours.

The patient underwent transurethral plasmakinetic resection of the prostate (TUPKP)to relieve urinary tract obstruction and obtain tissue for definitive pathological diagnosis. Intraoperatively, extensive bleeding was observed on the prostatic surface. A cauliflower-like tumor was also identified at the bladder neck, protruding into the bladder lumen. It showed invasion of both the bladder wall and the prostate. The procedure was uneventful, with an estimated blood loss of approximately 5 mL, and no blood transfusion was required. The resected prostatic tissue was sent for histopathological examination and immunohistochemical staining. The final diagnosis confirmed a malignant bladder tumor, specifically urothelial carcinoma (AJCC stage M1 IV). There was secondary malignant involvement of the prostate, along with metastases to the lungs and bones. After surgery, the patient experienced recurrent hematuria, which was attributed to postoperative surgical trauma. In response, intravenous cefotiam hydrochloride was initiated at a dose of 1.0 g every 8 hours as anti-infective therapy.

**Figure 1:**
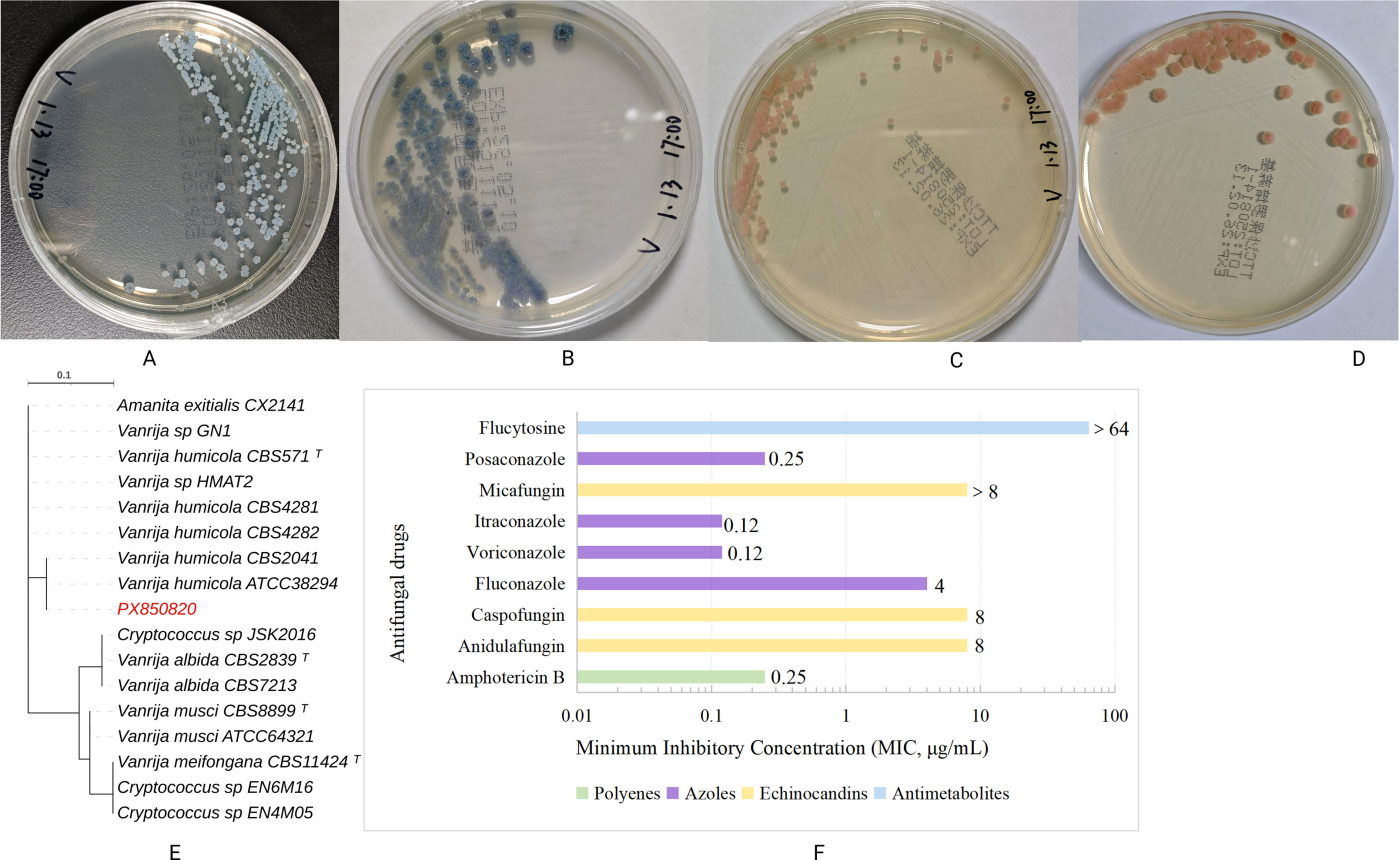
Microbiological identification of *V. humicola* and its antifungal susceptibility profile. **(A)** The patient-isolated fungus cultured on CHROMagar at 28 °C for 72 h. **(B)** The patient-isolated fungus cultured on CHROMagar at 28 °C for 168 h. **(C)** The patient-isolated fungus cultured on Sabouraud Dextrose Agar–TTC (SDA-TTC) solid medium at 28 °C for 72 h. **(D)** The patient-isolated fungus cultured on SDA-TTC solid medium at 28 °C for 168 h. **(E)** Maximum likelihood phylogenetic tree inferred from ITS sequences showing the relationship between the patient isolate (PX850820) and species of the genus *Vanrija*. The tree was constructed using the Kimura 2-parameter model with 1,000 bootstrap replicates. Type strains are indicated by the superscript “T”. *Amanita exititialis* CX2141 was used as the outgroup. **(F)** Antifungal susceptibility profile of the patient isolate. The color of each bar indicates the antifungal drug class, and the MIC value for each drug is shown above the corresponding bar.

During treatment, a urine sample was collected on October 9, 2025, due to persistent hematuria and pyuria, along with ongoing suspicion of UTI despite empirical antibiotic therapy (**Table 1**). This sample was sent for both bacterial and fungal culture before the initiation of radiotherapy and chemotherapy. Given the advanced stage of disease and persistent left hip pain, palliative radiotherapy was initiated on October 10, 2025. A total dose of 30 Gy was delivered in 10 fractions, targeting the bladder, prostate, and left pelvic bones. On the same day, systemic treatment with chemoimmunotherapy was also started. The regimen included toripalimab (240 mg), gemcitabine (0.6 g), and cisplatin (30 mg). The urine culture grew *Enterococcus faecalis* on October 10 at a concentration of 5 × 10^4^ CFU/mL. Fungal culture of the same urine specimen demonstrated heavy growth of an unidentified yeast (>10^5^ CFU/mL) on October 12. The fungus produced moderately sized pink, dry, and cerebriform with a fringed margin colonies after incubation on Sabouraud Dextrose Agar–TTC medium at 28 °C for 72 hours. For species identification, matrix-assisted laser desorption/ionization time-of-flight mass spectrometry (MALDI-TOF MS) was performed; however, it failed to identify the organism at the species level in our case. In view of persistent fever and elevated inflammatory markers, molecular identification was subsequently performed. Sequencing of the internal transcribed spacer (ITS) region, together with the D1–D2 domain of the large subunit ribosomal DNA, identified the isolate as *Vanrija humicola*. The corresponding ITS sequence was deposited in the GenBank database under accession number PX850820. Based on these findings, the patient was diagnosed with a mixed urinary tract infection caused by *Enterococcus faecalis* and *Vanrija humicola* (**Figure 1**).

**Table 1:** Laboratory results obtained at the time of clinical suspicion of urinary tract infection before antifungal therapy initiation.

Test category	Parameter	Result	Reference range	Unit
Hematology	WBCs	7.49	3.5~9.5	10^9^/L
	Neutrophil Percentage	83.1	40~75	%
	Lymphocyte	0.61	1.1~3.2	10^9^/L
	Hemoglobin	120	130~175	g/L
	Platelet	243	125~350	10^9^/L
Inflammatory Markers	CRP	95.1	0~6.0	mg/L
	Procalcitonin	0.16	0~0.5	ng/mL
Biochemistry	Glucose	4.91	3.9~6.1	mmol/L
	Direct Bilirubin	7.1	0~6.84	μmol/L
	ALT	9.2	9~50	U/L
	AST	14.6	15~40	U/L
	Urea	3.84	3.6~9.5	mmol/L
	Creatinine	84.5	57~111	μmol/L
Urinalysis	RBCs	67571	0~10	/μL
	WbcS	682	0~15	/μL
	Non-squamous Epithelial Cell	24	0~3	/μL
	Bacterium	69	0~33	/μL
	Occult Blood	3+	–	Cell/μL
	Leukocyte Esterase	2+	–	Cell/μL
Body Temperature	Peak (Tmax)	38.7	36.5~37.5	°C

WBCs, white blood cells; CRP, C-reactive protein; RBCs, red blood cells; ALT, alanine aminotransferase; AST, aspartate aminotransferase; Tmax, Maximum body temperature.

Following antimicrobial susceptibility testing and multidisciplinary consultation, treatment was initiated with intravenous amoxicillin–clavulanate at a dose of 1.2 g every 8 hours from October 13, 2025, to October 16, 2025. Intravenous voriconazole was administered for antifungal therapy, based on its low MIC value (0.12 µg/mL) observed in our isolate. It was given at a dose of 0.2 g every 12 hours. Intravenous voriconazole therapy was administered from October 14 to October 15, 2025, and was resumed from October 17 to October 20, 2025. Therapeutic drug monitoring of voriconazole plasma levels was not performed during treatment. All antibacterial and antifungal therapies were administered exclusively through the intravenous route, with no switch to oral therapy during hospitalization. Urine parameters were closely monitored, and repeat cultures were performed during therapy to assess microbiological response. During treatment, the patient developed several manageable adverse effects. These included nausea, vomiting, and grade II myelosuppression. All of these events were controlled effectively with supportive care.

After completing anticancer and antimicrobial therapy, urinary symptoms resolved, inflammatory markers normalized, and left hip pain improved. The patient was discharged on October 22, 2025.

## Discussion

We report a case of UTI caused by *V. humicola* and *E. faecalis* in a patient with UCB. Human infections caused by *V. humicola* remain extremely uncommon. Most reported cases occur in immunocompromised patients. The first case was described in 1975 in patients presenting with conjunctivitis and ophthalmic disease (15). In cancer patients, the first report was published in 1998 from Mexico City, describing four cases (16). Another report described a systemic infection in a seven-year-old boy from India with suspected leukemia or lymphoma, from whom isolates were recovered from both blood and urine (17). Reported clinical manifestations are predominantly urinary tract infections, including cases in cancer patients and in individuals with a history of multiple invasive procedures (**Table 2**) (14, 18). In contrast, *V. humicola* has also been detected in respiratory samples from four cystic fibrosis patients (20). These findings suggest that *V. humicola* may act as an opportunistic organism in healthcare settings, particularly among immunocompromised patients, rather than being solely an environmental contaminant.

**Table 2:** Summary of reported cases of *Vanrija humicola* infections, including patient demographics, underlying conditions, infection sites, clinical presentation, microbiological identification, MIC, antifungal therapy, and outcomes.

Author/year	Age/sex	Underlying condition/comorbidities	Site of infection	Clinical presentation	Microbiological identification	MIC (μg/mL)	Antifungal treatment	Outcome	Reference
Present Case/2025	82/M	Advanced urothelial carcinoma, chemo-immunotherapy	Urinary tract	Urinary frequency, urgency, dysuria, left hip pain	ITS and D1-D2 rDNA sequencing	AmB, 0.25; ANF, 8; CAS, 8; FLU, 4; VRC, 0.12; ITC, 0.12; MCF, >8; POS, 0.25; 5-FC >64.	Voriconazole IV	Discharged with symptoms resolved	This report
Jae Won Lee, et al./2024	46/F	Cervical cancer, past breast cancer/Ileostomy	Urinary tract	Hematuria	Bruker Biotyper MALDI-TOF	AmB, 0.25; ANF, 8; CAS, 8; FLU, 128; VRC, 2; ITC, 0.5; MCF, 8; POS, 1; 5-FC, 64.	None	Discharged with resolution of chief complaints	(14)
	49/M	Esophageal rupture with multiple repair surgery/Med iastinitis	Urinary tract	Not mentioned	Bruker Biotyper MALDI-TOF	AmB, 0.25; ANF, 8; CAS, 8; FLU, 64; VRC, 2; ITC, 0.25; MCF, 8; POS, 0.5; 5-FC, 64.	Voriconazole	Discharged with resolution of chief complaints	
	71/M	Non-small cell lung cancer, diabetes mellitus/Herpes simplex virus meningoencephalitis	Urinary tract	Hematuria	Bruker Biotyper MALDI-TOF	Not mentioned	None	Discharged with resolution of chief complaints	
	86/M	Unknown carcinoma on neck/Toxic epidermal necrolysis	Urinary tract	Not mentioned	Bruker Biotyper MALDI-TOF	Not mentioned	Caspofungin	Expired	
Lingning Meng, et al./2023	65	Chronic myeloid leukemia	Urinary tract	Not mentioned	18S rRNA	Not mentioned	Not mentioned	Not mentioned	(18)
Olender A/2025	26–32 (19)/f (n=4)	Cystic fibrosis	Lung	Not mentioned	Bruker Biotyper MALDI-TOF	AmB, 0.19~0.75; ANF, 0.03~0.75; CAS, 0.05~0.38; FLU, 2~32; VRC, 0.05~32; ITC, 0.5~32; MCF, 0.02~0.06; POS, 0.5~32.	Not mentioned	Not mentioned	(20)
Shinde SM et al/2004	7 (M)	Suspected leukemia/lymphoma	Systematic	Dyspnea, tachycardia, generalized LAD, bilateral pedal edema, hepatosplenomegaly, rales/rhonchi	Mini API and ID 32 strip biochemical reactions	Not mentioned	Amphotericin B, fluconazole	Discharged with resolution of chief complaints	(17)

M, male; F, female; AmB, Amphotericin B; ANF, Anidulafungin; CAS, Caspofungin; FLU, Fluconazole; VRC, Voriconazole; ITC, Itraconazole; MCF, Micafungin; POS, Posaconazole; 5-FC, 5-flucytosine.

Opportunistic fungal infections are an emerging concern in cancer patients. Several major risk factors have been identified, including prolonged neutropenia, intensive chemotherapy, corticosteroid therapy, disruption of mucosal barriers, prolonged antibiotic exposure, and hospital-related factors such as invasive procedures (21–25). In the present case, multiple factors may have contributed to the development of opportunistic fungal infection. First, advanced urothelial carcinoma itself may disrupt the urinary tract mucosal barrier and compromise host immunity. Second, the patient underwent TUPKP, and postoperative hematuria was observed. We speculate that the invasive nature of the surgical procedure may have damaged the urinary tract mucosa. Postoperatively, the patient received a three-lumen urethral catheter and a drainage tube, which also created favorable conditions for fungal infection. Subsequently, the patient received chemotherapy and immunotherapy, which may have resulted in myelosuppression and consequently weakened the host’s immune defense. In addition, advanced age, empirical broad-spectrum antibiotics, and concomitant bacterial infection may act as additional contributing factors for fungal colonization and infection. Bacterial infection can alter the urinary microbial ecosystem, potentially creating conditions that allow opportunistic fungi to proliferate. Our case supports the observation that disruption of the urinary mucosal barrier and changes in the local microbial ecological niche may create an environment for colonization and infection of rare environmental yeasts (14). From a pathobiological perspective, the urinary microbiome and mycobiome are increasingly recognized as playing important roles in the development of urothelial cancer and modulating responses to treatment (26).

An important consideration in the present case was differentiating infection from colonization. The rare environmental yeasts recovered from urine may occasionally represent transient contamination or asymptomatic colonization (27). However, in our case, several findings supported clinically significant infection rather than colonization. These included persistent urinary symptoms, fever, marked pyuria, elevated inflammatory markers, heavy fungal growth (>10^5^ CFU/mL) in urine culture in the setting of advanced malignancy, recent urinary tract surgery, chemo-immunotherapy, and prolonged hospitalization. In addition, the patient showed clinical improvement after initiation of voriconazole therapy, with resolution of urinary symptoms and normalization of inflammatory markers. These overall clinical and microbiological findings suggested that *V. humicola* acted as an opportunistic urinary pathogen in the current case.

In both clinical and laboratory settings, accurate identification of rare fungi remains challenging. The accuracy of phenotypic identification methods ranges from approximately 63.3% to 76.7% (19). MALDI-TOF-MS has emerged as a powerful tool for the identification of rare fungi, with reported identification accuracy exceeding 90%; however, its performance is highly dependent on the coverage of the reference database (28). A study from Korea reported successful identification of *V. humicola* using the Bruker Biotyper MALDI-TOF MS system, indicating that accurate identification is achievable when the reference database is sufficiently populated (14). Nevertheless, identification of *V. humicola* and other rare fungi by MALDI-TOF-MS remains variable and may fail in settings with limited database representation. The ITS region, which has been designated by mycologists as the universal DNA barcode for fungi, plays a critical role in species-level identification (29). In the present case, molecular sequencing of the ITS region and the D1-D2 domain of the large subunit rDNA, followed by phylogenetic analysis, was essential for accurate species identification. Previous studies have suggested that using MALDI-TOF-MS or updated high-throughput platforms as rapid identification tools is useful. Sequencing (ITS/D1-D2) can then be used for further confirmation, which may be the best practice for identifying rare fungal isolates (30–33). Furthermore, because it can be misidentified as non-neoformans *Cryptococcus* or *Candida* species, special caution is required. Whenever a yeast shows phenotypic characteristics inconsistent with patient symptoms or antifungal susceptibility testing (AST), molecular identification should be prioritized (34).

Rare yeasts often exhibit elevated minimum inhibitory concentrations (MICs) to azoles and/or echinocandins, whereas the activity of amphotericin B generally remains relatively stable (35–38). Many rare yeasts, including *V. humicola*, currently have no breakpoints defined by either the Clinical and Laboratory Standards Institute (CLSI) or the European Committee on Antimicrobial Susceptibility Testing (EUCAST). Therefore, antifungal therapy should be guided by both species identification and MIC values, rather than relying only on empirical treatment, because antifungal susceptibility can vary widely among different taxa (39, 40). In the present case, AST was performed before definitive species identification. The isolate demonstrated elevated MIC values for echinocandins but lower MICs for azoles and amphotericin B. The MIC against amphotericin B was <1 mg/L, consistent with previously reported studies (14, 20). The elevated MIC to echinocandins in our case is similar to the study reported from Korea, but it is different from the four isolates reported from sputum samples in Poland, which had MICs of <1 mg/L in all isolates (14, 20). Regarding azole drugs, the current strain had lower MICs than previously reported strains. For fluconazole, the MIC of the current strain was 4 mg/L, while it was >64 mg/L in strains reported in Korea. In the strains isolated in Poland, one isolate had an MIC of 2 mg/L, and the other three had MICs of >32 mg/L. Similarly, the voriconazole MIC of the strains from Korea was 2 mg/L, while the strains from Poland had MICs of <1 mg/L for two isolates and >32 mg/L for the other two (14, 20). In our case, the MIC for voriconazole was 0.12 mg/L, indicating good *in vitro* susceptibility. In the present case, amphotericin B was not favored because of its known nephrotoxicity and infusion-related adverse effects, which are of particular concern in elderly patients with advanced malignancy and multiple comorbidities (41, 42). Although itraconazole and posaconazole are active against some yeasts, their oral absorption and plasma exposure may be variable, and their intravenous formulations have limitations in routine initial use (43, 44). Fluconazole showed higher MIC values than voriconazole in this isolate, suggesting weaker *in vitro* activity. By contrast, voriconazole has predictable intravenous administration, established use in invasive fungal infections, and a much lower MIC in this case. Therefore, voriconazole therapy was started, and the patient responded well to the treatment, highlighting the importance of MIC testing for timely and appropriate antifungal therapy. In previously reported cases, two infected patients were treated with voriconazole and caspofungin, respectively (14). The current case, together with previous reports, suggests that because MIC values differ among cases, antifungal susceptibility-guided therapy should be prioritized for the treatment of *V. humicola* infection. However, patient risk factors, infection site, and the distinction between colonization and invasive disease should also be considered when selecting therapy. Empirical treatment with an azole or amphotericin B may be reasonable while awaiting definitive laboratory results.

Furthermore, this study also reinforces that rare environmental fungi can cause clinically significant infections in immunocompromised patients, particularly when intensive medical care and invasive procedures are involved. We agree that *V. humicola* should be regarded as a potential nosocomial urinary tract pathogen, while also recognizing that it may act as a urinary colonizer. Further investigations integrating comprehensive clinical characterization with high-resolution molecular approaches are warranted. Such studies may help determine whether *V. humicola* contributes directly to cancer-related processes or primarily acts as an opportunistic pathogen or colonizer in immunocompromised hosts.

## Conclusion

This case, together with the literature review, demonstrates that environmental fungi like *V. humicola* can cause opportunistic infections in severely immunocompromised cancer patients. Timely identification and confirmation of rare pathogens using molecular methods are important for accurate diagnosis. The current isolate and literature indicate that *V. humicola* has high MICs to echinocandins, low MICs (<1) to amphotericin B, and variable MICs to azoles. AST and MIC-guided therapy are essential to improve clinical outcomes. Future molecular studies with detailed clinical data are needed to determine whether *V. humicola* affects cancer-related processes or just represents an environmental contaminant.

## Data Availability

The gene sequence data from this study have been deposited at the NCBI GenBank database with the accession number PX850820.

## References

[1] Bray F Laversanne M Sung H Ferlay J Siegel RL Soerjomataram I . Global cancer statistics 2022: GLOBOCAN estimates of incidence and mortality worldwide for 36 cancers in 185 countries. CA: A Cancer J For Clin. (2024) 74:229–63. doi: 10.3322/caac.21834 38572751

[2] Lobo N Afferi L Moschini M Mostafid H Porten S Psutka SP . Epidemiology, screening, and prevention of bladder cancer. Eur Urol Oncol. (2022) 5:628–39. doi: 10.1016/j.euo.2022.10.003 36333236

[3] Su X Tao Y Chen F Han X Xue L . Trends in the global, regional, and national burden of bladder cancer from 1990 to 2021: an observational study from the global burden of disease study 2021. Sci Rep. (2025) 15:7655. doi: 10.1038/s41598-025-92033-5 40038504 PMC11880295

[4] Lin HJ Hu RM Chen HC Lin CC Lee CY Chou CY . CA125 for the diagnosis of advanced urothelial carcinoma of the bladder: a systematic review and meta-analysis. Cancers. (2023) 15:813. doi: 10.3390/cancers15030813 36765770 PMC9913454

[5] Rani B Ignatz-Hoover JJ Rana PS Driscoll JJ . Current and emerging strategies to treat urothelial carcinoma. Cancers. (2023) 15:4886. doi: 10.3390/cancers15194886 37835580 PMC10571746

[6] Lin S Jiang Y Xu T Zhou X Bai X Liu S . The incidence and risk factors of urinary tract infections in patients undergoing bladder tumor resection: a systematic review and meta-analysis. BMC Urol. (2025) 25:267. doi: 10.1186/s12894-025-01751-5 41146151 PMC12557927

[7] Fadhil R . Urinary tract infection among patients with bladder cancer : Bacteriological studies. Iraqi J Cancer Med Genet. (2018) 5. doi: 10.29409/ijcmg.v5i2.92

[8] Abdul-Lateef Y . The association of urinary tract infection with urothelial carcinoma. Iraqi J Cancer Med Genet. (2018) 8. doi: 10.29409/ijcmg.v8i2.156

[9] Zhang W Zhang H Gao Y Lei J Suo C . Fungi and cancer: unveiling the complex role of fungal infections in tumor biology and therapeutic resistance. Front Cell Infect Microbiol. (2025) 15:1596688. doi: 10.3389/fcimb.2025.1596688 40557321 PMC12185418

[10] Bilal H Khan MN Khan S Shafiq M Fang W Zeng Y . Fungal influences on cancer initiation, progression, and response to treatment. Cancer Res. (2025) 85:413–23. doi: 10.1158/0008-5472.can-24-1609 39589783

[11] Puerta-Alcalde P Monzo-Gallo P Aguilar-Guisado M Ramos JC Laporte-Amargos J MaChado M . Breakthrough invasive fungal infection among patients with haematologic Malignancies: a national, prospective, and multicentre study. J Infect. (2023) 87:46–53. doi: 10.1016/j.jinf.2023.05.005 37201859

[12] Pereira MI Marques G Nascimento T Cortesão E Tomé R Geraldes C . Rare invasive fungal infections in hematologic patients: a large unicentric series. Blood. (2023) 142:5871. doi: 10.1182/blood-2023-188856

[13] Imanishi D Abe K Kera Y Takahashi S . Draft genome sequence of the yeast Vanrija humicola (formerly Cryptococcus humicola) strain UJ1, a producer of d-aspartate oxidase. Genome Announce. (2018) 6:e00068-18. doi: 10.1128/genomea.00068-18 29545290 PMC5854778

[14] Lee JW Won EJ Sung H Kim M-N . Emergence of Vanrija humicola as a pathogen of urinary tract infections in Korea. Ann Clin Microbiol. (2024) 27:31–7. doi: 10.5145/acm.2024.27.1.5

[15] Nitzulescu V Niculescu M . Cryptococcus species isolated from an ocular lesion. Arch Roumaines Pathol Experiment Microbiol. (1975) 34:363–5. 1227446

[16] Alvarez Gasca MA Argüero Licea B Pliego Castañeda A García Tena S . Fungal agents isolated from cancer patients. Rev Latinoamericana Microbiol. (1998) 40:15–24. 10932730

[17] Shinde SM Vanarse KS Pandit AN . Systemic humicolus cryptococcosis. Indian Pediatr. (2004) 41:1162–4. doi: 10.1007/978-981-16-4047-6_17 15591670

[18] Meng L Liu C Zhu L Wang X Li C . Draft genome sequence of yeast Vanrija sp. strain TS01, isolated from leukemia patient's urine. Microbiol Resour Announce. (2023) 12:e0015223. doi: 10.1128/MRA.00152-23 PMC10508090 37610212

[19] Belgacem S Chebil W Ben Salem S Babba O Mastouri M Babba H . Identification and antifungal susceptibility profile of uncommon yeast species at Fattouma Bourguiba University Hospital in Tunisia. Med Mycol. (2024) 62:myae070. doi: 10.1093/mmy/myae070 38986508

[20] Olender A Bogut A Dąbrowski W Pietrzak DJ Szukała M Wójtowicz-Bobin M . Analysis of antifungal drug resistance among Candida spp. and other pathogenic yeasts isolated from patients in Eastern Poland: diagnostic problems. Infect Drug Resist. (2025) 18:2187–99. doi: 10.2147/idr.s504516 40321598 PMC12049117

[21] Domínguez C Enciso LJ Cuervo SI Rondón MA Espinel CF . D-index as a risk factor for invasive fungal infections in patients with acute lymphoblastic leukemia from a reference hematology center in Bogota. Cureus. (2023) 15:e38612. doi: 10.7759/cureus.38612 37288185 PMC10243397

[22] Johnston DL Lewis V Yanofsky R Gillmeister B Ethier MC Mitchell D . Invasive fungal infections in paediatric acute myeloid leukaemia. Mycoses. (2013) 56:482–7. doi: 10.1111/myc.12063 23437849

[23] Dassi N Cappellano AM da Silva A da Silva NS Carlesse F . Invasive fungal infections in pediatric patients with central nervous system tumors: novel insights for prophylactic treatments? Front Oncol. (2023) 13:1248082. doi: 10.3389/fonc.2023.1248082 37965468 PMC10641464

[24] Roumani AM Benmouffok N . Risk factors for invasive fungal disease in pediatric oncology. Int J Clin Res. (2022) 3:167–72. doi: 10.38179/ijcr.v3i1.202

[25] Moraitaki E Kyriakidis I Pelagiadis I Katzilakis N Stratigaki M Chamilos G . Epidemiology of invasive fungal diseases: a 10-year experience in a tertiary pediatric hematology–oncology department in Greece. J Fungi. (2024) 10:498. doi: 10.3390/jof10070498 39057383 PMC11278103

[26] Heidar NA Bhat TA Shabir U Hussein AA . The urinary microbiome and bladder cancer. Life Bsl Switz. (2023) 13:812. doi: 10.3390/life13030812 36983967 PMC10053959

[27] Duan X Zhai Z Sun L Li H Zheng S Li X . A review of case reports of rare clinical yeast infections in the last five years. Mycopathologia. (2025) 190:56. doi: 10.1007/s11046-025-00962-6 40540156

[28] Dutkiewicz M Garros M Bui J Charlier V Da Silva E Lemaire M . Comparison of MALDI-TOF MS instruments and databases for the identification of uncommon yeasts, Aspergillus spp. and rare filamentous fungi. J Clin Microbiol. (2025) 63:e0161224. doi: 10.1128/jcm.01612-24 40372046 PMC12153315

[29] Schoch CL Seifert KA Huhndorf S Robert V Spouge JL Levesque CA . Nuclear ribosomal internal transcribed spacer (ITS) region as a universal DNA barcode marker for fungi. Proc Natl Acad Sci. (2012) 109:6241–6. doi: 10.1073/pnas.1117018109 22454494 PMC3341068

[30] Raja HA Miller AN Pearce CJ Oberlies NH . Fungal identification using molecular tools: a primer for the natural products research community. J Nat Prod. (2017) 80:756–71. doi: 10.1021/acs.jnatprod.6b01085 28199101 PMC5368684

[31] Gabaldón T . Recent trends in molecular diagnostics of yeast infections: from PCR to NGS. FEMS Microbiol Rev. (2019) 43:517–47. doi: 10.1093/femsre/fuz015 PMC8038933 31158289

[32] Wickes BL Wiederhold NP . Molecular diagnostics in medical mycology. Nat Commun. (2018) 9:5135. doi: 10.1038/s41467-018-07556-5 30510235 PMC6277409

[33] Morovati H Kord M Ahmadikia K Eslami S Hemmatzadeh M Kurdestani KM . A comprehensive review of identification methods for pathogenic yeasts: challenges and approaches. Adv BioMed Res. (2023) 12:187. doi: 10.4103/abr.abr_375_22 37694259 PMC10492613

[34] Desnos-Ollivier M Lortholary O Bretagne S Dromer F . Azole susceptibility profiles of more than 9,000 clinical yeast isolates belonging to 40 common and rare species. Antimicrob Agents Chemother. (2021) 65:e02615-20. doi: 10.1128/aac.02615-20 33820766 PMC8315974

[35] Jabrodini A Eghtedarnejad E Ghanbarzadeh A Motamedi M Jafari M Kharazi M . Molecular identification and antifungal susceptibility profile of rare and emerging yeast species causing onychomycosis. BMC Res Notes. (2025) 18:167. doi: 10.1186/s13104-025-07197-0 40229655 PMC11998136

[36] Turan D Habip Z Odabaşı H Dömbekçi E Gündoğuş N Özmen M . Antifungal susceptibilities of rare yeast isolates. J Fungi Bsl Switz. (2025) 11:645. doi: 10.3390/jof11090645 41003191 PMC12470942

[37] Stavrou AA Pérez-Hansen A Lackner M Lass-Flörl C Boekhout T . Elevated minimum inhibitory concentrations to antifungal drugs prevail in 14 rare species of candidemia-causing Saccharomycotina yeasts. Med Mycol. (2020) 58:987–95. doi: 10.1093/mmy/myaa005 32043147

[38] Borman AM Muller J Walsh-Quantick J Szekely A Patterson Z Palmer MD . MIC distributions for amphotericin B, fluconazole, itraconazole, voriconazole, flucytosine and anidulafungin and 35 uncommon pathogenic yeast species from the UK determined using the CLSI broth microdilution method. J Antimicrob Chemother. (2020) 75:1194–205. doi: 10.1093/jac/dkz568 32025716

[39] Espinel-Ingroff A Cantón E Pemán J . Antifungal resistance among less prevalent Candida non-albicans and other yeasts versus established and under development agents: a literature review. J Fungi Bsl Switz. (2021) 7:24. doi: 10.3390/jof7010024 33406771 PMC7824324

[40] Franconi I Lupetti A . *In vitro* susceptibility tests in the context of antifungal resistance: beyond minimum inhibitory concentration in Candida spp. J Fungi Bsl Switz. (2023) 9:1188. doi: 10.3390/jof9121188 38132789 PMC10744879

[41] Maertens J Birne R Felton T Neofytos D Hoenigl M . Liposomal amphotericin B and renal safety: review of the evidence and clinical considerations. J Antimicrob Chemother. (2026) 81:dkaf473. doi: 10.1093/jac/dkaf473 41549663 PMC12813295

[42] Stanzani M Vianelli N Cavo M Maritati A Morotti M Lewis Russell E . Retrospective cohort analysis of liposomal amphotericin B nephrotoxicity in patients with hematological Malignancies. Antimicrob Agents Chemother. (2017) 61:e02651-16. doi: 10.1128/aac.02651-16 28607011 PMC5571305

[43] Abuhelwa AY Foster DJR Mudge S Hayes D Upton RN . Population pharmacokinetic modeling of itraconazole and hydroxyitraconazole for oral SUBA-itraconazole and Sporanox capsule formulations in healthy subjects in fed and fasted states. Antimicrob Agents Chemother. (2015) 59:5681–96. doi: 10.1128/aac.00973-15 26149987 PMC4538523

[44] Chen L Krekels EHJ Verweij PE Buil JB Knibbe CAJ Brüggemann RJM . Pharmacokinetics and pharmacodynamics of posaconazole. Drugs. (2020) 80:671–95. doi: 10.1007/s40265-020-01306-y 32323222 PMC7183491

