# Correction: Urinary tract infection caused by rare yeast *Vanrija humicola* in a urothelial carcinoma patient: a case report and literature review

**DOI:** 10.3389/fimmu.2026.1905301

**Published:** 2026-06-16

**Authors:** Hanman Qiu, Hong Du, Xiaohui Li, Xunsong Wang, Yan Li, Hazrat Bilal, Bin Xu

**Affiliations:** 1 School of Public Health, Jiangxi Medical College, Nanchang University, Nanchang, Jiangxi, China; 2 Jiangxi Key Laboratory of Oncology (2024SSY06041), Jiangxi Cancer Hospital & Institute, The Second Affiliated Hospital of Nanchang Medical College, Nanchang, Jiangxi, China; 3 Department of Pharmacy, Qingdao Mental Health Center, Qingdao, Shandong, China; 4 Jiangxi Key Laboratory of Translational Cancer Research, Jiangxi Cancer Hospital, The Second Affiliated Hospital of Nanchang Medical College, Jiangxi Cancer Institute, Nanchang, Jiangxi, China

**Keywords:** antifungal susceptibility testing, case report, opportunistic rare fungal infection, urothelial carcinoma, *Vanrija humicola*

The **Ethics Statement** was erroneously given as “The authors take full responsibility for the work, ensuring that any concerns about accuracy or integrity are properly addressed. All procedures followed ethical guidelines set by the institutional or national research committee(s) and complied with the Declaration of Helsinki (2013 revision). Institutional approval for publication was obtained from the Human Research Ethics Committee of the Jiangxi Cancer Hospital and Institute (Approval No. 2026ky018). The requirement for written informed consent was waived by the Institutional Ethics Committee because no identifiable patient information is included in this case report. Written informed consent was obtained from the participant/patient(s) for the publication of this case report”.

The correct **Ethics Statement** is “The authors take full responsibility for the work, ensuring that any concerns about accuracy or integrity are properly addressed. All procedures followed ethical guidelines set by the institutional or national research committee(s) and complied with the Declaration of Helsinki (2013 revision). Institutional approval for publication was obtained from the Human Research Ethics Committee of the Jiangxi Cancer Hospital and Institute (Approval No. 2026ky018). Written informed consent was obtained from the participant/patient(s) for the publication of this case report”.

The original version of this article has been updated.

